# Cost-utility analysis of total knee arthroplasty alone and in comparison with post-surgical rehabilitation and conservative treatment in the Republic of Kazakhstan

**DOI:** 10.1186/s12962-022-00379-8

**Published:** 2022-09-01

**Authors:** Dinara Serikova-Esengeldina, Natalya Glushkova, Gulzada Abdushukurova, Akmaral Mussakhanova, Ainur Mukhamejanova, Zaituna Khismetova, Dmitry Bokov, Alexandr Ivankov, Maiya Goremykina, Yuliya Semenova

**Affiliations:** 1 Department of Public Health, Semey Medical University, Semey, Kazakhstan; 2 Faculty of Medicine and Healthcare, Al-Farabi Kazakh National University, Almaty, Kazakhstan; 3 Institute of Higher Medical Postgraduate Education, Akhmet Yassawi University, Turkestan, Kazakhstan; 4 Department of Public Health and Management, Astana Medical University, Nur-Sultan, Kazakhstan; 5 Department of Family Medicine #2, Astana Medical University, Nur-Sultan, Kazakhstan; 6 Institute of Pharmacy, Sechenov First Moscow State Medical University, Moscow, Russia; 7 Laboratory of Food Chemistry, Federal Research Center of Nutrition, Biotechnology and Food Safety, Moscow, Russia; 8 Independent Researcher, Almaty, Kazakhstan; 9 Department of Rheumatology and Noncommunicable Diseases, Semey Medical University, Semey, Kazakhstan; 10 School of Medicine, Nazarbayev University, Nur-Sultan, Kazakhstan

**Keywords:** Total knee arthroplasty, Cost analysis, QALY, Cost-utility ratio, Incremental cost-effectiveness ratio

## Abstract

**Background:**

Despite ample international knowledge on cost-effectiveness of total knee arthroplasty (TKA), it has never been a subject of investigation in Kazakhstan or other post-Soviet economies. Our study aimed to carry-out the cost-utility analysis of TKA alone and in comparison with post-surgical rehabilitation and conservative treatment at health care facilities of Kazakhstan.

**Methods:**

Two hundred and forty four patients with knee osteoarthritis (KOA) who underwent TKA in orthopedic departments of Almaty, Nur-Sultan and Semey hospitals between January 1, 2019 and September 30, 2019 were followed-up for 12 months. The health-related quality of life was measured by the EQ-5D utility and Western Ontario and McMaster Universities Osteoarthritis Index was used to measure the patients’ health status. The costs were estimated from the view of health care provider. We calculated the cost per QALY, the Cost-Utility Ratio and the Incremental Cost-Effectiveness Ratio.

**Results:**

At the time of 12-month follow-up patients who received TKA alone or with the course of rehabilitation showed benefit over patients from the group of conservative treatment in terms of overall health status. Mean QALY gained at 12 months constituted 1.66 for the group that received TKA with rehabilitation, 1.48 for the group that received TKA alone and 0.24 for the group that received conservative treatment. Mean cost per QALY gained was USD 30 795.75 for KOA patients under conservative treatment, USD 6 323.69 for KOA patients subjected to TKA and USD 2 670.32 for KOA patients with rehabilitation course after TKA.

**Conclusion:**

Both TKA and TKA with rehabilitation could be considered as highly cost-effective interventions. The data obtained could be of interest for policy makers, medical professionals and KOA patients.

## Background

Nowadays, total knee arthroplasty (TKA) is considered to be a highly effective procedure for patients with end-stage osteoarthritis. This surgery enables a significant reduction in symptoms (particularly pain) and restoration of functions in a large proportion of patients. It also proved to be safe since the rate of complications is rather small [1]. As the prevalence of osteoarthritis increases with age, more and more patients will face the need for TKA over the next decades. Such, it was projected that by 2050, the incidence rate of primary TKA will increase to 299 per 100,000 population (43% growth) due to enlarged number of surgeries performed on male patients, with the highest modeled increase observed in patients aged 50-65 years [2]. In 2010, hip and knee osteoarthritis together were ranked as the 11th contributor to global disability according to the Global Burden of Disease study [3].

A number of international studies devoted to the economic evaluation of TKA in comparison with non-surgical approach proved its cost-effectiveness and ability to improve the patients’ quality of life [4]. According to the findings of the Knee Arthroplasty Trial, mean quality of life improved from 0∙39 pre-operatively to 0∙71 1 year postoperatively and was subjected only to gradual decline thereafter [5]. Still, there is variation in costs and benefits of TKA in dependence with patient subgroups: the surgery is more cost-effective in younger patients, in those presenting with more severe symptoms preoperatively, and also in those operated in high-volume medical centers. Even if delaying TKA till older age may appear to be cost-saving in a short term, this is not a cost-effective strategy in a long term [6]. Although obese and comorbid patients carry higher medical costs, the surgery remains to be cost-effective based on the threshold established by the National Institute for Health and Clinical Excellence (NICE): £20 000–£30 000 per QALY gained [7].

Annually, the rate of knee osteoarthritis (KOA) in the Republic of Kazakhstan (hereafter—Kazakhstan) increases year by year, constituting 1.1% growth [8]. Partly, this may be attributed to a high prevalence of Vitamin D deficiency [9]. Still, TKA is not widely available across the country’s provinces as it is predominantly performed in metropolises (Almaty and Nur-Sultan). This is due to the lack of medical facilities and qualified personnel but also because of a high cost, which is an important obstacle for regional medical centers. However, within the course of national health care reforms, the country’s government made a particular attention to the construction and equipment of regional health care centers [10]. Thus, the issue of broad implementation of TKA at regional medical centers has to be solved in Kazakhstan to make this procedure more accessible to patients residing outside metropolises.

Despite ample international knowledge on cost-effectiveness of TKA, it has never been a subject of investigation in Kazakhstan—a Central-Asian post-Soviet country. Similarly, it was never a matter of research in other post-Soviet economies (Russian Federation, Kyrgyzstan, Uzbekistan, Tajikistan, Turkmenistan), which share many features in functioning and budgeting of health care systems. Meanwhile, the results of cost-effectiveness studies conducted in other countries may not be applicable to Kazakhstan, taking into account the differences in health policies with non-Soviet style economies. Therefore, our study aimed to carry-out the cost-utility analysis of TKA alone and in comparison with post-surgical rehabilitation and conservative treatment at health care facilities of the Kazakhstan Republic.

## Materials and methods

### Patients under study

The study comprised the data of 269 patients entering scheduled primary TKA in orthopedic departments of Almaty, Nur-Sultan and Semey hospitals between January 1, 2019 and September 30, 2019. The patients were invited to participate in the study and to fill in the EQ-5D and the WOMAC questionnaires. At the time of their follow-up visits, which took place approximately 6 and 12 months after the surgery was performed, the patients were asked to fill in the same questionnaires. Since 25 patients refused to participate or returned the questionnaires with missing data, the complete data were available for 244 patients (90.7% response rate). All patients gave written informed consent to participate.

The data on demographic and clinical characteristics of patients under study are presented in Table 1. The rehabilitation following TKA included postoperative exercise program under nurse supervision. Those patients who received the course of rehabilitation were older and had monthly income above the median average that constituted 112,200 Tenge in 2019, which is an equivalent for USD 291.84 as of December 20, 2019 [11]. There was a significant difference in mean duration of KOA between the study groups with maximum duration observed in the group with post-surgical rehabilitation. Also, the same group had the highest rate of disability at baseline (71.7%) as compared with other groups under study. None of the patients in TKA group required revision after 1 year of follow-up in contrast with the patients who received the course of rehabilitation (p < 0.001).

**Table 1:** Demographic and clinical characteristics of patients under study

Variable	Type of intervention (n = 244)						Test of difference*	
	Conservative treatment, n = 122		TKA, n = 62		TKA with rehabilitation, n = 60			
	N	%	N	%	N	%	χ2	p-value
Sex								
Females	90	73.8	44	71.0	53	88.3	6.258	0.044
Males	32	26.2	18	29.0	7	11.7		
Age (mean ± SD), years*	65.05	3.19	64.66	3.01	66.95	2.77	10.394	0.000
Ethnicity								
Kazakhs	20	16.4	20	32.3	18	30.0	7.414	0.025
Russians	102	83.6	42	67.7	42	70.0		
Education								
Secondary	4	3.3	10	16.1	19	31.7	106.066	0.000
Vocational secondary	20	16.4	22	35.5	41	68.3		
Higher	98	80.3	30	48.4	0	0.0		
Monthly income								
Below the median range	78	63.9	30	48.4	0	0.0	67.212	0.000
Above the median range	44	36.1	32	51.6	60	100.0		
Duration of osteoarthritis (mean ± SD), years*	9.69	1.93	11.58	1.27	12.28	2.04	48.552	0.000
Disability								
Absent	48	39.3	50	80.6	17	28.3	39.424	0.000
Present	74	60.7	12	19.4	43	71.7		
Need for TKA revision								
Absent	–	–	50	100.0	23	62.2	22.547	0.000
Present	–	–	0	0.0	14	37.8		

^*^ANOVA was test of difference for quantitative variables

*SD* Standard deviation, *TKA* Total knee arthroplasty

Before initiation of the data collection, we received the approval of Ethics Committee of Semey Medical University (Protocol #7, dated 30.05.2017).

### Treatments provided

In Kazakhstan, provision of care to KOA patients is regulated by the standards of care, approved by the Expert Committee of the Republican Centre for Health Development (RCHD). Such, the standards of conservative treatment and medical rehabilitation are envisaged by the document entitled “Protocol for clinical diagnosis and treatment of KOA [12], while TKA is performed in accordance with the “Knee Replacement Surgery Protocol” [13]. The same documents specify indications and contraindications to each type of treatment. Since healthcare budgeting is grounded on these regulatory documents, the composition of study groups was strongly dependent on the requirements set by the above referenced treatment standards. Thus, the inclusion criteria for this study were as follows: (i) KOA stages III and IV; (ii) being adult (older than 18 years of age); (iii) having no psychiatric disorder with cognitive deficit; (iv) giving informed consent to participate in the study.

### Questionnaires

The health-related quality of life (HRQoL) was measured by the EQ-5D utility – a standardized generic health status measurement [14]. The EQ-5D utility has five dimensions (5Ds), which are: mobility, self-care, usual activities, pain/discomfort, and anxiety/depression and the patients are expected to self-rate the severity level for each dimension. In turn, the severity levels are rated on a 5-point scale, ranging from level 1 (no problems) to level 5 (extreme problems). Visual analogue scale (EQ-VAS) was used to assess the patient’s health status on the day of the interview. For this purpose, the KOA patients were asked to mark their health status on a 20-cantimeter vertical scale with end points of 0 (worst health that could be imagined) and 100 (best health that could be imagined). All patients filled the EQ-5D at baseline, at 6 months following treatment and then at 12 months afterwards. The area under the utility curve was used to calculate the number of quality-adjusted life years (QALYs) accrued by each patient following the treatment provided.

In addition to EQ-5D, we utilized the Western Ontario and McMaster Universities Osteoarthritis Index (WOMAC)—a standardized tool used to measure the health status of patients with osteoarthritis, including pain, stiffness, and functioning of joints. The WOMAC has five items for pain, which range from 0 to 20, two items for stiffness, which range from 0 to 8, and 17 items for physical function (range 0–68) [15]. Like in case with EQ-5D, all patients self-administered the WOMAC at baseline, at six and then at 12 months following treatment.

### Cost-utility analysis

We took the healthcare payer’s perspective when assessing all costs and excluded personal and social services from the analysis, although TKA has a potential to extend the longevity of paid employment and to reduce the costs related to personal care. Since the majority of TKA-associated expenditures occur during the in-patient period [1], we obtained the data on direct hospital costs from National Health Insurance Foundation, which is a single public agency in Kazakhstan responsible for healthcare financing. It stores the data on all treatment costs, including postoperative patient visits and admissions.

We calculated QALY scores to assess TKA effect. For this, we used the following formulas with account for life expectancy in Kazakhstan, which in 2019 was equal to 73.13 years [16]:

Initial QALY = Initial EQ-5D score*Patient Life expectancy (73.13—Exact age at the study moment).

Final QALY = EQ-5D score*Patient Life expectancy (73.13—Exact age at the study moment).

QALY gained = Final QALY—Initial QALY.

Cost per QALY = QALY gained/Cost of treatment.

Cost per QALY (3% discount) = QALY gained/(Cost of treatment + (Cost of treatment*0.03)).

In addition, we analyzed separately various factors affecting cost (age, sex, stage of KOA, body mass index (BMI), place of treatment and need for revision TKA).

We also calculated the Cost-Utility Ratio (CUR) = total cost/total effect (USD/QALY).

The Incremental Cost-Effectiveness Ratio (ICER) for the patients from TKA group was calculated on the basis of the following formula:

ICER = (cost of TKA—cost of conservative therapy)/(QALY for TKA—QALY for conservative therapy).

Meanwhile, for the group that received TKA with rehabilitation, ICER was calculated as follows:

ICER = (cost of TKA with rehabilitation—cost of TKA) / (QALY for TKA with rehabilitation—QALY for TKA).

All expenditures were expressed in USD (USD 1 = 384.46 Kazakhstan Tenge as of December, 20, 2019) [11].

### Statistical analyses

Study data were analyzed with the help of SPSS for Windows statistical software version 20.0 (license of Semey Medical University). The results were presented as mean and standard deviation (SD) for quantitative variables with a pattern of distribution close to normal, or as absolute numbers and percentages for qualitative variables. The statistical significance of differences was analyzed with Student’s paired T-test or with Pearson’s χ2 test. One-way analysis of variance (ANOVA) test was utilized to compare means of more than two study groups, while multivariate analysis of variance (MANOVA) was used to compare two or more vectors of means. We considered the p-values less than 0.05 as statistically significant.

## Results

**Fig. 1:**
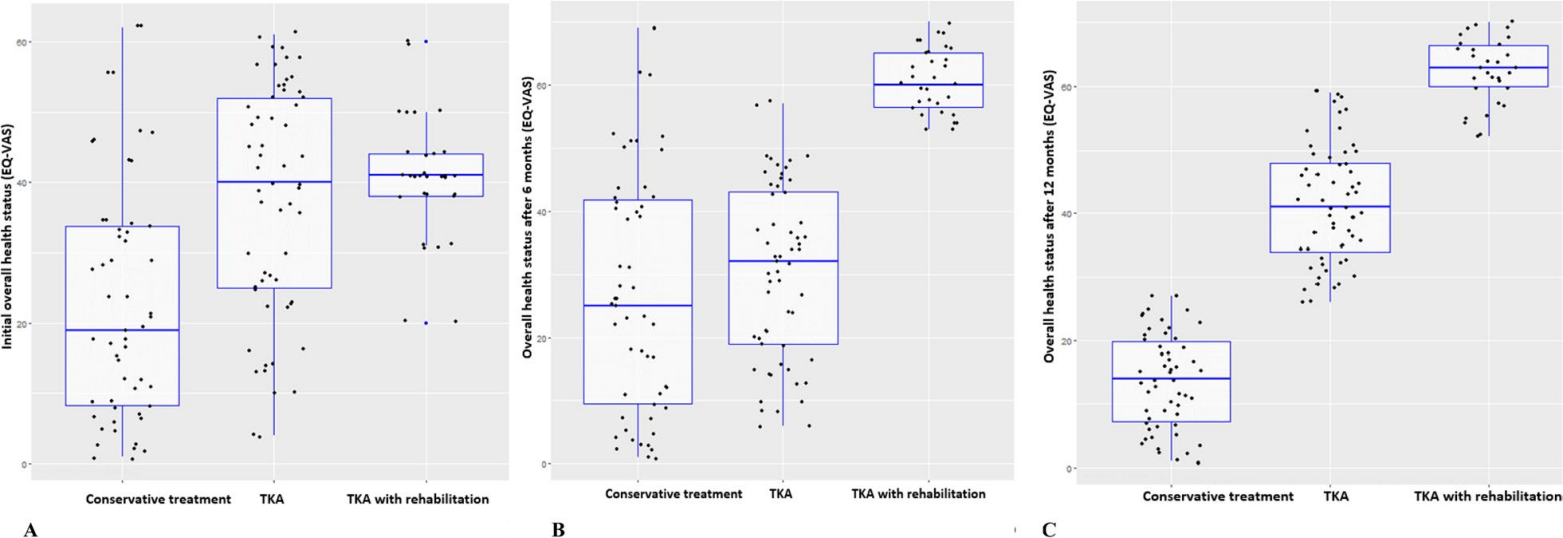
Overall health status using the visual analogue scale (EQ-VAS) at baseline (**A**), at 6-month follow-up (**B**) and at 12-month follow-up (**C**)

Table 2 presents the subscales of the WOMAC in KOA patients at different study periods. At baseline, there was a significant difference between the study groups in stiffness, which was more pronounced in TKA group. Besides, those patients who received TKA with rehabilitation had higher global score, indicating the presence of worse pain, stiffness, and decrease in overall physical functioning. Meanwhile, this situation was different both at the time of 6-month and 12-month follow ups: patients who received conservative treatment had significantly higher scores for each of the WOMAC scales. Moreover, these scores tended to deteriorate at 12-month follow up in comparison with 6-month assessment, probably indicating the natural progression of KOA. According to Fig. 1, those patients who received TKA with rehabilitation, had better overall health status based on the visual analogue scale already at the time of 6-month follow-up. At the time of 12-month follow-up patients from both surgical groups showed benefit over patients from the group of conservative treatment in terms of overall health status.

**Table 2:** The Western Ontario and McMaster Universities Osteoarthritis Index (WOMAC) subscales in patients under study

Variable	Type of intervention						Test of difference	
	Conservative treatment, n = 122		TKA, n = 62		TKA with rehabilitation, n = 60		ANOVA	
	N	%	N	%	N	%	F	p-value
At baseline								
Pain	17.35	4.43	17.36	1.84	17.13	2.71	0.091	0.913
Stiffness	4.18	1.25	4.71	1.23	4.09	1.26	4.851	0.009
Physical function	63.58	5.78	64.54	4.06	62.60	4.66	2.193	0.114
Global score	80.35	3.99	80.76	2.06	83.38	1.83	19.457	0.000
6 months follow-up								
Pain	14.19	3.60	9.22	1.28	4.76	1.00	261.243	0.000
Stiffness	3.11	0.92	1.96	0.83	1.54	0.38	92.221	0.000
Physical function	51.25	6.93	37.39	3.68	15.61	1.19	920.249	0.000
Global score	55.90	3.68	33.61	3.53	20.64	3.70	2080.636	0.000
12 months follow-up								
Pain	14.47	1.80	7.77	2.24	2.55	1.23	929.351	0.000
Stiffness	4.31	1.16	1.95	0.46	0.53	0.09	433.882	0.000
Physical function	55.88	3.00	26.54	3.57	4.54	1.18	7014.082	0.000
Global score	59.96	2.89	26.78	3.23	11.97	2.45	6418.663	0.000

*TKA* Total knee arthroplasty

On a scale of 0 to 100, the mean EQ-5D utility scores significantly improved in patients from both surgical groups. As for the group with conservative treatment, the EQ-5D scores improved at 6 months but deteriorated at 12 months following treatment, although this difference did not reach the level of statistical significance. The mean hospital costs at 12 months were the highest for the patients receiving conservative treatment (USD 5905.79), followed by TKA (USD 5156.50) and TKA with rehabilitation (USD 4479.44). Mean QALY gained at 12 months constituted 1.66 for the group that received TKA with rehabilitation, 1.48 for the group that received TKA alone and 0.24 for the group that received conservative treatment. Mean cost per QALY gained was USD 30 795.75 for KOA patients under conservative treatment, USD 6323.69 for KOA patients subjected to TKA and USD 2670.32 for KOA patients with rehabilitation course after TKA. The cost-utility ratio of conservative treatment was USD 4187.01/QALY, while that for TKA and TKA with rehabilitation was USD 3914.25/QALY and USD 3400.30/QALY, respectively. Both TKA and TKA with the course of rehabilitation could be considered as cost-saving procedures since ICER took negative values (Table 3).

**Table 3:** EQ-5D and cost data

Variable	Type of intervention						Test of difference:	
	Conservative treatment, n = 122		TKA, n = 62		TKA with rehabilitation, n = 60		ANOVA	
	Mean	SD	Mean	SD	Mean	SD	F	p-value
Mean EQ-5D at baseline	59.88	4.77	63.26	3.76	63.93	1.95	26.837	0.000
Mean EQ-5D at 6 months after treatment	70.09	7.73	71.67	4.64	85.61	3.21	135.034	0.000
Mean EQ-5D at 12 months after treatment	63.04	4.15	80.38	5.28	91.07	2.63	995.675	0.000
EQ-5D improved at 12 months, p-value*	0.523		< 0.001		< 0.001			
Mean hospital costs at 12 months, USD	5905.79	343.75	5156.50	422.56	4479.44	311.94		
Life expectancy in 2019 = 73.13 years	8.08	3.19	8.47	3.01	6.18	2.77	10.3945	0.000
Mean QALYs gained at 12 months	0.24	0.28	1.48	0.83	1.66	0.70	162.44508	0.000
Mean QALYs gained at 12 months (discounted by 3%)	0.23	0.27	1.43	0.80	1.61	0.68	162.84544	0.000
Cost per QALY, USD	30795.75	18657.38	6323.69	13316.57	2670.32	1735.35	96.418735	0.000
Cost per QALY, USD (discounted by 3%)	31719.62	19217.10	6513.40	13716.06	2750.43	1787.41	96.418727	0.000
CUR	4187.01	549.82	3914.25	320.76	3400.30	236.73	65.000559	0.000
ICER			−606.32		−3581.42			

^*^The Paired Samples t-Test

*CUR* Cost-Utility Ratio, *ICER* incremental Cost-Effectiveness Ratio, *SD* Standard Deviation, *TKA* Total Knee Arthroplasty, *QALY* Quality-adjusted Life Years, *USD* United States Dollars

The cost per QALY gained for different subgroups of patients is shown in Table 4. In the TKA group the cost per QALY gained of older patients was higher than that of younger patients, while the mean QALY was lower. Surgical patients with stage 4 KOA benefited more than stage 3 KOA patients: they had higher mean QALY. Still, the same patients had higher cost per QALY gained. Provision of surgery at the level of regional health care center resulted in higher mean QALY both in the group of TKA and in the group of TKA with rehabilitation. Nevertheless, TKA patients operated at the level of the regional health care center had higher cost per QALY gained as compared with those operated in one of the metropolis health clinics. As for the group of TKA with rehabilitation, it was quite the opposite: the cost per QALY gained was higher for those patients, who were operated at the level of metropolis health care center.

**Table 4:** Estimated QALYs for different patient subgroups

Variable	Type of intervention												Test of difference for QALY gained: MANOVA		Test of difference for Cost per QALY: MANOVA	
	Conservative treatment				TKA				TKA with rehabilitation							
	n = 122				n = 62				n = 60							
	QALY gained		Cost per QALY		QALY gained		Cost per QALY		QALY gained		Cost per QALY					
	Mean	SD	Mean	SD	Mean	SD	Mean	SD	Mean	SD	Mean	SD	F	p-value	F	p-value
Age, years																
≥ 65	0.28	0.36	26561.49	13661.86	1.75	0.77	5756.39	15493.72	2.43	0.34	1469.48	240.33	99.888	< 0.001	55.017	< 0.001
< 65	0.19	0.14	36127.79	22512.10	0.81	0.53	7710.43	5122.23	1.28	0.49	3270.74	1848.88				
Gender																
Male	0.27	0.32	30369.68	20907.10	1.51	0.84	6668.43	15421.07	1.57	0.68	2832.22	1782.86	58.329	< 0.001	37.233	< 0.001
Female	0.15	0.06	31994.08	10088.39	1.40	0.81	5481.00	5807.60	2.39	0.41	1444.48	282.74				
Stage of osteoarthritis																
Stage 3	0.25	0.35	31731.98	17469.50	1.47	0.78	4294.71	4041.79	1.64	0.80	2879.81	2007.67	0.018	0.893	0.632	0.428
Stage 4	0.22	0.17	29535.44	20252.43	1.51	1.04	14,777.76	28529.46	1.74	0.39	2140.42	328.10				
BMI																
< 25.0	0.26	0.34	30901.42	18140.63	1.34	0.91	5615.30	4981.50	1.95	0.58	1918.60	518.39	3.007	0.053	1.949	0.146
25.0–29.9					1.35	0.45	3114.80	959.90	1.85	0.00	1775.40	0.00				
≥ 30.0	0.22	0.18	30632.85	19621.27	1.72	0.85	8975.59	21651.69	1.08	0.62	4315.06	2266.39				
Place of treatment																
Metropolis					1.42	0.79	7442.21	16567.13	1.62	0.91	4297.67	3083.06	0.937	0.336	0.015	0.901
Regional					1.59	0.89	9922.52	20369.16	1.80	0.50	2646.72	625.88				
Revision TKA																
Absent					1.47	0.78	4294.71	4041.79	1.36	0.85	3776.41	2375.60	n/a	n/a	n/a	n/a
Present									1.77	0.59	2017.91	527.04				

*BMI* Body Mass Index, *TKA* Total Knee Arthroplasty, *QALY* Quality-adjusted Life Years, *USD* United States Dollars

## Discussion

This study aimed to conduct the cost-utility analysis of TKA alone, TKA with rehabilitation and conservative treatment at health care facilities of Kazakhstan to enable better understanding of local health economics. In agreement with earlier published reports [17, 18], both TKA and TKA with rehabilitation have proven to be cost-effective. To the best of our knowledge, this study is the first to report on pharmacoeconomics of TKA in a post-Semashko health care system. Besides, there is lack of international publications investigating cost-effectiveness or cost-utility of TKA in combination with the course of post-surgical rehabilitation.

A number of international guidelines set thresholds for health care expenditures to enable an optimal choice of cost-effective interventions. Such, the World Health Organization (WHO) launched the “Choosing Interventions that are Cost-Effective” (CHOICE) initiative to help the countries decide on their health care priorities. According to this project, the cost per QALY gained is compared with the country’s gross-domestic product (GDP) and an intervention is considered to be highly cost-effective if it does not exceed the GDP per capita, cost-effective if it constitutes the 1–3 GDP per capita and not cost effective if it exceeds the GDP per capita more than 3 times [19]. Our study showed that mean cost of TKA was USD 6323.69 per QALY gained, while that of TKA with rehabilitation was USD 2670.32. As in 2019 the national GDP per capita was USD 9812.39 [20], both TKA and TKA with rehabilitation are a highly cost-effective interventions.

Still, in 2019 the Kazakhstani budget for medical care was 3.1% of the national GBP. In 2018 per capita health spending constituted USD 278.5 and out-of-pocket expenditures composed 38.5% of total health expenditures or 1.2% of GBP [21]. TKA is provided for the government expense to the country’s citizens and there are waiting lists for surgery in many health care centers. Patients who are not willing to wait can get TKA surgery in the private sector but the cost is prohibitively high for most of them. However, the cost of rehabilitation course is commonly not covered by the government’s funds and this was the reason why it was only provided to a part of patients in our study since not everybody could afford it.

Of interest is the fact that in the USA many osteoarthritis patients are ready to pay out-of-pocket nearly half of the total costs for TKA (USD 12 797) [22]. In Canada patients are willing to co-pay USD 3 378 in average to increase the longevity of their joint implant [23]. In Australia 70% of patients are willing to pay something and 18% of patients are willing to pay the actual average cost of TKA, which is approximately 15 000 Australian Dollars [24]. This might not be applicable to the realities of Kazakhstan, where many older people are not willing to pay anything for health care services as it was not practiced in the soviet health care system. Nevertheless, in established market economies patients value joint replacement surgeries and are willing to spend a substantial amount of money out-of-pocket to get TKA. This willingness correlates with patient risk taking and spending habits [25].

Physical therapist management is an integral part of rehabilitation course for patients undergoing TKA. In this regard, a number of strategies have been developed: preoperative and/or postoperative exercise program with or without patient education, training for recovery of motor functions, neuromuscular electrical stimulation, cryotherapy, etc. These approaches have different levels of certainty according to a clinical practice guideline on TKA developed by the American Physical Therapy volunteer guideline development group [26]. Nevertheless, there is an agreement that patients undergoing TKA should receive a preoperative exercise program, which helps to achieve better functional outcomes after surgery. Although preoperative education on postoperative rehabilitation program, use of assistive devices, prevention of falls and overall planning of post-operative care is not adequately supported by the published evidence [27], it is considered to be important and desirable.

Encouragement of early mobility with gradual progression of physical activities, including both aerobic and weight-bearing exercises is crucial for prompt recovery and restoration of physical function. The typical training course should involve balance interventions and exercises to improve walking, range of motion and movement symmetry [26]. Neuromuscular electrical stimulation is applied in addition to physical exercises. It helps to improve walking, stair-climbing and to strengthen quadriceps and hamstrings. However, it is not universally available and may be costly [28]. As for cryotherapy, it is commonly used for postoperative pain management and its benefits include low cost and ease of application [29]. In Kazakhstan, a typical rehabilitation course after TKA mostly covers postoperative exercise program, which is provided under supervision of a specially trained nurse and includes patient education. Still, this course is paid out-of-pocket and the cost is unaffordable for the patients from underprivileged socio-economic classes.

This study has certain limitations and relatively small sample size obtained is one of them. Another limitation is that we estimated costs from the view of health care provider although patients undergoing TKA are in need for other community services, like transportation and assistance in activities of daily living. However, we believe that hospital costs make up the bulk of TKA costs. Also, we compared the benefits of TKA with conservative treatment and the on-the-ground assumption was that health-related quality of life does not change over time, which is not always right since conservative treatment might alleviate the symptoms of KOA and improve HRQoL. Finally, we followed-up the patients only up to 1 year, which might not be sufficient for tracking remote treatment outcomes.

## Conclusions

TKA alone and TKA with the course of post-surgical rehabilitation are highly cost-effective interventions, although costs and benefits vary for different categories of patients and the costs might be prohibitively high for certain groups of patients in the Republic of Kazakhstan. Thus, these data could be used to initiate a dialog with policymakers on expansion of the list of free medical services. Also, our results could be of interest for TKA candidates regarding the expected outcomes and may assist medical professionals in appropriate selection of patients.

## Data Availability

The data and material are available from the corresponding author on request.

## Abbreviations

ANOVA: Analysis of variance
CUR: Cost-utility ratio
GBP: Great Britain Pound
GDP: Gross-domestic product
ICER: Incremental cost-utility ratio
HRQoL: Health-related quality of life
KOA: Knee osteoarthritis
MANOVA: Multivariate analysis of variance
NICE: National Institute for Health and Clinical Excellence
QALY: Quality-adjusted life years
TKA: Total knee arthroplasty
USD: United States Dollar
VAS: Visual analogue scale
WOMAC: Western Ontario and McMaster Universities Arthrose index
